# Asymptomatic carriers of coronavirus disease 2019 among healthcare workers in Isfahan, Iran

**DOI:** 10.2217/fvl-2020-0224

**Published:** 2021-01-28

**Authors:** Hamed Fakhim, Elahe Nasri, Shima Aboutalebian, Sahar Gholipour, Mahnaz Nikaeen, Afsane Vaezi, Somayeh Mousavi, Sama Faramarzi, Armin Farhang, Shaghayegh Haghjooy Javanmard, Mehrdad Salahi, Ali Darakhshandeh, Kazem Ahmadikia, Hossein Mirhendi

**Affiliations:** 1 ^1^Infectious Diseases & Tropical Medicine Research Center, Isfahan University of Medical Sciences, Isfahan, Iran; 2 ^2^Core Research Facilities (CRF), Isfahan University of Medical Sciences, Isfahan, Iran; 3 ^3^Department of Medical Parasitology & Mycology, School of Medicine, Isfahan University of Medical Sciences, Isfahan, Iran; 4 ^4^Department of Environmental Health Engineering, School of Health, Isfahan University of Medical Sciences, Isfahan, Iran; 5 ^5^Department of Medical Laboratory Science, School of Allied Medical Sciences, Iran University of Medical Sciences, Tehran, Iran; 6 ^6^Department of Pharmaceutical Biotechnology, School of Pharmacy and Pharmaceutical Sciences, Isfahan University of Medical Sciences, Isfahan, Iran; 7 ^7^Applied Physiology Research Center, Cardiovascular Research Institute, Isfahan University of Medical Science, Isfahan, Iran; 8 ^8^Department of Internal Medicine, School of Medicine, Isfahan University of Medical Sciences, Isfahan, Iran; 9 ^9^Department of Medical Parasitology & Mycology, School of Public Health, Tehran University of Medical Sciences, Tehran, Iran

**Keywords:** asymptomatic carrier, COVID-19, healthcare workers, Iran

## Abstract

**Aim:** This study aimed to investigate the prevalence of coronavirus disease 2019 (COVID-19) among healthcare workers (HCWs) in Isfahan, Iran. **Materials & methods:** HCWs in COVID-19 wards of three referral COVID-19 hospitals in Isfahan were screened and tested for COVID-19 infection. **Results:** In total, 102 HCWs were screened whose median age was 43 years old. Moreover, 21 (20.5%) of them had a history of suspected infection with SARS-CoV2, mostly (66.6%) without any symptoms while six (28.5%) of them suffered from relatively mild diseases and one (4.7%) was diagnosed with pulmonary embolism. **Conclusion:** It was found that HCWs were prone to be asymptomatic carriers while their computed tomography images were normal. Therefore, it is recommended that reverse-transcriptase real-time-PCR be essential for the diagnosis of infections.

The coronavirus disease 2019 (COVID-19) recently caused an outbreak of viral pneumonia in China that quickly spread to many countries [1,2]. The global mortality rate differs in various areas, nations and different types of patients [3,4]. Iran was one of the countries affected by the worldwide outbreak of COVID-19. Currently, it is difficult to estimate the overall mortality rate due to uncertainties regarding the testing policies of the different countries and also the rapidly changing dynamics of the disease [5,6]. Clinical presentation varies widely from a mild common cold-like illness to severe viral pneumonia leading to acute respiratory distress syndrome that is often fatal [1]. The person-to-person transmission as well as transmission through direct contact with contaminated surfaces have been confirmed. However, the transmission of COVID-19 infection from asymptomatic carriers and mildly symptomatic patients with normal chest computed tomography (CT) is a recent finding [4,7]. It is essential to protect healthcare workers (HCWs) from asymptomatic carriers and mildly symptomatic patients of COVID-19 to prevent onward transmission to their patients and colleagues. Targeted infection prevention and control measures may decrease the risk of healthcare-associated outbreaks [8–11]. This could be even more challenging, considering the poor access to personal protective equipment in different countries [8]. There is a need for extensive adoption of an expanded screening strategy for asymptomatic carriers and mildly symptomatic HCWs in order to limit the transmission of the virus within a hospital and from hospitals to the community. The present study aimed to investigate COVID-19 among HCWs in three referral hospitals in Isfahan, Iran. In this regard, all published papers on this topic were reviewed based on country, number of participants and positive cases.

## Materials & methods

This cross-sectional study with follow-up was conducted in three referral hospitals in Isfahan, namely 166-bed Omid Hospital, 224-bed Khorshid Hospital and 114-bed Isaabne-Maryam Hospital. The HCWs who worked in COVID-19 wards voluntarily tested for COVID-19 infection according to the local infection control procedure from 20 February to 15 March 2020. For the purposes of the study, nasopharyngeal samples were taken from 102 randomly selected HCWs. The RNA was extracted by using a viral RNA isolation kit (ROJE, Yazd, Iran) according to the instructions of the manufacturer. Reverse-transcriptase real-time (rRT-PCR) targeting the N and RdRp genes (Pishtaz Teb kit, Tehran, Iran) was performed. The amplification was performed with a cycle of 20 min at 50°C for reverse transcription and 3 min at 95°C primary denaturation followed by 45 cycles at 94°C (10 s) and 55°C (40 s) by a Light Cycler 96-well system (Roche, Germany). According to the protocol of the manufacturer, CT < 40 in both genes was considered positive. All samples and experiments were processed at the referral clinical laboratory for COVID-19 of Isfahan University of Medical Sciences, Isfahan, Iran. The recorded data included demographic characteristics and current or previous signs and symptoms. For each positive rRT-PCR case, CT was performed and the virus clearance was defined as negatives of nucleic acid tests. This study was approved by the Research and Ethics Committee (IR.MUI.RESEARCH.REC.1398.776) of Isfahan University of Medical Sciences. Furthermore, written informed consent was obtained from the next of kin of the participants for the publication of this report.

## Results

In total, 102 HCWs were screened, 21 (20.5%) of whom were infected with SARS-CoV2. The subjects had a median age of 43 years old (within a range of 26 to 51 years old) and 69 (67.6%) of them were female. Most HCWs with COVID-19 had no symptoms (66.6%) and six (28.5%) of them suffered from a relatively mild disease while one (4.7%) was diagnosed with pulmonary embolism (PTE). Furthermore, seven (6.8%), two (9.5%) and one (4.7%) HCWs had a fever, cough and diarrhea, respectively, without lymphopenia and leukopenia during the course of illness. None of the subjects had a history of traveling or contact with known or suspected COVID-19 cases. In total, the chest CT images of two (9.5%) cases showed typical findings, namely ground-glass or patchy shadows in the lungs, while the CT scans of the rest of the cases were normal. Local protocol for COVID-19 was followed, and asymptomatic HCWs were isolated at home for two weeks. The HCWs with relatively mild disease were treated with hydroxychloroquine sulfate oral consumption of 400 mg twice a day (BID) for 24 h followed by 200 mg BID and HCW with PTE was treated with daily consumption of 2.5 mg warfarin. None of the cases developed severe pneumonia, and none of them died. In total, 20 cases (75.0%) after the 2-week follow-up were cured (two continuous negatives of nucleic acid tests) among whom only one asymptomatic case was positive. Nevertheless, for this case, the result of nasopharyngeal swab specimen was negative for 2019-nCoV after two weeks. The rRT-PCR Ct value range for N and RdRp genes were 24.35–38.5 and 28.5–37.5, respectively. The Ct values tended to be higher in HCWs who were tested later in the course of infection. The Ct values were similar for HCWs with and without any symptoms on the day of screening.

## Discussion

Through the quick establishment of an expanded HCW screening strategy in Isfahan, it was discovered that 66.6% of the participants tested positive for severe acute respiratory syndrome coronavirus 2 (SARS-CoV-2), while they did not have symptoms. These cases were asymptomatic, compared with those previously reported in Wuhan, China [1,2]. Only one of the cases was diagnosed with PTE and only six cases suffered from a relatively mild disease during the study. Similar to previous studies, fever, cough and diarrhea were the main symptoms [9,10]. According to the systematic review and meta-analysis about HCWs as asymptomatic carriers, fever, anosmia and myalgia were the main associated factors of SARS-CoV-2 infection; besides, 40% of subjects did not report any symptoms of COVID-19 during the screening, and 0.5% of the infected HCWs died [12]. Lymphopenia and leukopenia, formerly associated with the disease severity [1,2,9,10], were uncommon in the subjects of the present research. Moreover, it was found that HCWs were more likely to be asymptomatic and have normal CT images. Therefore, it is recommended to make rRT-PCR testing essential for the diagnosis of asymptomatic infections among HCWs. These outcomes indicate that asymptomatic HCWs can cause person-to-person transmission and should be considered a source of infection in hospital wards to prevent potential outbreaks and the infection of different types of patients [13,14]. Folgueira *et al.* in their study found that there were no significant differences among the infection rates of HCWs working in low-, intermediate- and high-risk exposure settings [15]. In the present study, the comparison did not show a statistically significant difference between the presumably high- and low-risk exposure settings. Only few studies are available from different countries about HCWs as asymptomatic carriers (Table 1). According to statistics, the frequency of positive tests among asymptomatic workers in our study population (66.6%) was similar to those of Spain (50%) and the UK (57%) [16,17]. It is noteworthy that, Italy, the USA and China reported rates of 100% [18–20]. A mathematical model that considered asymptomatic carriers indicated that the initial value of the effective reproduction number could range from 5.5 to 25.4 which is in agreement with the results of this research [14]. Rivett *et al.* reported that 3% of HCWs in the asymptomatic screening group tested positive for SARS-CoV-2 in a large UK teaching hospital. Moreover, they found that 57% of them were asymptomatic/paucisymptomatic, while 40% of them had experienced symptoms; this finding was in line with those of this study [17]. Furthermore, based on the results of a study conducted in a tertiary Hospital in Wuhan, 93 out of 6574 non-first-line HCWs (1.4%) were infected with COVID-19 [21]. This result is also consistent with those of the present research. In this study, the prevalence of subclinical infection was 0.74% among asymptomatic first-line HCWs and 1% among non-first-line HCWs. Standard national guidelines were followed for asymptomatic cases that required isolation for 10 days after the test. However, the isolation of HCWs who developed symptoms after a positive swab test was extended for 14 days after symptom onset. Consequently, we recommend that in a setting with limited testing capacity, a high priority should be given to HCWs screening programs. This is essential for the prevention of staff-to-staff or staff-to-patient transmission; otherwise, it can lead to extensive morbidity and mortality in vulnerable patients [14,21–25]. Epidemiological data will also clarify whether the hospital staff is more likely to be infected in the community or at work. This investigation also had some limitations. First, the study population was relatively small. Second, the study was conducted in only one region of Iran and it may not be able to reflect the spread of infection throughout the whole country. Third, this study was carried out only on HCWs who were exposed to SARS-CoV-2; therefore, the results cannot be expected to represent all HCWs. This report was the first to investigate the asymptomatic carriers of COVID-19 among HCWs in Iran. It should be noted that the recent strategy in Iran is that people with moderate and mild respiratory symptoms should isolate themselves and their family members.

**Table 1.** Frequency of asymptomatic positive samples in studies conducted on the prevalence of coronavirus disease 2019 in healthcare workers.

Study (year)	Participants	n of positive cases	Asymptomatic positives/total positives (percent)	Country	Ref.
Fusco *et al.* (2020)	115	4	4/4 (100%)	Italy	[18]
Olalla *et al.* (2020)	498	2	1/2 (50%)	Spain	[16]
Vahidy *et al.* (2020)	2872	112	112/112 (100%)	USA	[19]
Graham *et al.* (2020)	464	160	70/160 (44%)	UK	[26]
Brandstetter *et al.* (2020)	201	36	2/36 (5%)	Germany	[27]
Shields *et al.* (2020)	554	13	13/13 (100%)	UK	[28]
Ma *et al .* (2020)	33	4	No information	China	[29]
Lombardi *et al*. (2020)	1573	139	17/139 (12%)	Italy	[30]
Lan *et al*. (2020)	592	83	2/61 (2%)	USA	[31]
Lahner *et al*. (2020)	2057	58	18/58 (31%)	Italy	[32]
Houlihan *et al*. (2020)	200	42	34/42 (80%)	UK	[33]
Bhattacharya *et al*. (2020)	106	24	No information	India	[34]
Borras-Bermejo *et al*. (2020)	2655	403	225/403 (56%)	Spain	[35]
Antonio-Villa *et al*. (2020)	35095	11226	341/11226 (3%)	Mexico	[36]
Khalil *et al.* (2020)	266	47	16/47 (34%)	UK	[37]
Lai *et al.* (2020)	335	3	3/3 (100%)	China	[20]
Roxby *et al.* (2020)	62	2	2/2 (100%)	USA	[38]
Guery *et al.* (2020)	136	3	1/3 (33%)	France	[39]
Keeley *et al.* (2020)	1533	282	0/288 (0%)	UK	[11]
Rivett *et al.* (2020)	1032	30	17/30 (57%)	UK	[17]
Current study (2020)	102	21	14/21 (66%)	Iran	

## Conclusion

Results of this study provided data about HCWs who were asymptomatic carriers of COVID-19 infections. It was found that HCWs were likely to be asymptomatic with normal CT image results; therefore, it is recommended that rRT-PCR be made essential for the diagnosis of infections. Further investigation is needed about the mechanism by which the asymptomatic carriers can acquire and transmit COVID-19. The collected data can be used to improve national public health guidelines.

**Summary points**

- In Isfahan, Iran, 21 (20.5%) healthcare workers had a history of suspected infection with SARS-CoV2.
- It is recommended to make reverse-transcriptase real-time-PCR test for the monitoring of healthcare workers.
- Further epidemiological studies are needed to clarify whether hospital staff is more likely to be infected in the community or at work.
